# An intermediate term benefits and complications of gamma knife surgery in management of glomus jugulare tumor

**DOI:** 10.1186/s12957-016-0779-7

**Published:** 2016-02-15

**Authors:** Raef F. A. Hafez, Magad S. Morgan, Osama M. Fahmy

**Affiliations:** Department of Neurosurgery and Gamma Knife center, International Medical Center (IMC), Cairo, Egypt

**Keywords:** Gamma knife surgery, Gamma knife complications, Glomus jugulare tumor, Stereotactic radiosurgery

## Abstract

Glomus tumors are rare skull base slow-growing, hypervascular neoplasms that frequently involve critical neurovascular structures, and delay in diagnosis is frequent. Surgical removal is rarely radical and is usually associated with morbidity or mortality. Gamma knife surgery (GKS) has gained an increasing dependable role in the management of glomus jugulare tumors, with high rate of tumor growth control, preserving or improving clinical status and with limited complications. This study aims to evaluate intermediate term benefits and complications of gamma knife surgery in management of twenty-two patients bearing growing glomus jugulare tumors at the International Medical Center (IMC), Cairo, Egypt, between 2005 and 2011. The mean follow-up period was 56 months (range 36–108 months); there were 3 males, 19 females; mean age was 43.6 years; 15 patients had GKS as the primary treatment; 2 patients had surgical residuals; 2 had previous radiation therapy; and 3 previously underwent endovascular embolization. The average tumor volume was 7.26 cm^3^, and the mean marginal dose was 14.7 Gy*.* Post gamma knife surgery through the follow-up period neurological status was improved in 12 patients, 7 showed stable clinical condition and 3 patients developed new moderate deficits. Tumor volume post GKS was unchanged in 13 patients, decreased in 8, and showed tumor regrowth in 1 patient. Tumor progression-free survival in our studied patients was 95.5 % at 5 and 7 years of the follow-up period post GKS***.*** Gamma knife surgery could be used safely and effectively with limited complications as a primary management tool in the treatment of glomus jugulare tumors controlling tumor growth with preserving or improving clinical status especially those who do not have significant cranial or cervical extension, elderly, and surgically unfit patients; moreover, it is safe and highly effective as adjuvant therapy as well.

## Background

Glomus tumors are rare skull base benign slow-growing, highly vascular locally invasive tumors, also known as paragangliomas or chemodectomas. They originate from preganglionic tissues that can be found in the adventitia of the jugular bulb (glomus jugulare). These rare tumors reportedly occur predominantly in women, in a ratio of 1:1,000,000, in the sixth and seventh decade of life [1–5].

Although mostly benign and slow-growing, glomus jugulare tumors have a high propensity for local invasion of adjacent vascular structures, lower cranial nerves, middle ear, petrous apex, cavernous sinus, inner ear, carotid canal, or even up to infratemporal fossa, which may result in substantial morbidity and often complicate the surgical management of these tumors. The operative approach is complicated by the fact that lesions may be both intradural and extradural even with cervical components, with engulfment of critical neurovascular structures. Thus, it is not surprising that resection entails a great deal of morbidity or mortality and leaves behind usually large residual tumors [2, 4–7].

The most common symptoms are pulsatile tinnitus, conductive deafness, hypoacusis, headache, vertigo, and multiple cranial nerve palsy with possible involvement of 5th to 12th cranial nerves. The neurological symptoms are related to the region affected by the tumor. This can infiltrate neurovascular structures, the temporal bone, the jugular foramen, the hypoglossal canal, the clivus, the cavernous sinus, and cervical region [1, 2, 5–8].

Cranial nerve involvement produces hoarseness and dysphagia. The presence of jugular foramen syndrome (paresis of cranial nerves IX–XI) is pathognomonic for this tumor, but it usually follows the initial symptoms of decreasing hearing and pulsatile tinnitus. Less commonly, glomus tumors produce facial nerve palsy, hypoglossal nerve palsy, or Horner syndrome. Ataxia and brain stem symptoms may infrequently develop [2, 9–12, 16].

Treatment strategies for glomus jugulare tumors include microsurgery, preoperative embolization followed by surgical resection, fractionated external beam radiotherapy, and radiosurgery in the form of gamma knife radiosurgery. The use of various procedures may be the result of planned staging, or procedures may be used in combination as a salvage treatment after recurrence or progression. Although glomus cells are radio-resistant yet, radiotherapy helps decrease tumor growth by inducing fibrosis around the supplying vessels (endarteritis obliterance) [12, 13, 15].

Gamma knife surgery has been proposed as an alternative to conventional external beam radiotherapy as it has the capability of delivering precise high-dose radiation to a small localized field increasing the chances of obliterative endarteritis while reducing complications by sparing adjacent normal structures [10, 12, 14, 15].

## Material and method

### Objective

This study aims to evaluate an intermediate term benefits and complications of gamma knife surgery in the treatment of glomus jugulare tumors and its role as a primary modality of treatment.

### Method

A retrospective review was undertaken of the results obtained in 22 patients harboring glomus jugulare tumors that underwent gamma knife surgery (GKS) for recurrent, residual, or unrespectable tumors at the International Medical Center (IMC), Cairo, Egypt, from beginning of 2005 and end of 2011. The median clinical and radiological follow-up period was 56 months ranged (36–108 months). All patients underwent complete neurological assessment, examination before the treatment with MRI and audiograms. Follow-up includes clinical neurological evaluation and MRI brain with contrast. The follow-up post gamma knife done regularly each 6 months in the first year then yearly afterward.

In our studied 22 patients with glomus jugulare tumors,15 patients underwent gamma knife surgery as primary treatment and used as adjuvant therapy for 7 patients. In those treated with gamma knife as adjuvant therapy, 2 had previous partial microsurgical tumor removal with sizable residuals, another 2 had previous treatment with fractionated radiation therapy (typical 45–50 Gy) with regrowth of their tumors with in 12 and 18 months. Another 3 patients had endovascular embolization prior to GKS for their large hypervascular tumor volumes (19.4, 16, and 14.2 cm^3^), as preparation for microsurgery where two of them surgically operated before GKS leaving sizable residuals and one presented directly to GKS.

### Radiosurgery technique

The Elekta Leksell gamma knife version B model used before the beginning of 2010 then the 4-C version used afterward for the treatment of studied 22 patients with glomus jugulare tumors. The stereotactic frame was placed as low as posterior and as much to the side of the lesion with the head in a position of flexion. Target localization was achieved using MRI with contrast performed with T1 axial and coronal-weighted sequence at 2-mm slice thickness on zero angle without gap. T1-fat subtraction and also T2 axial sequence were also used to eliminate tumor edema, bone, fat, and embolized material if used. Treatment planning performed using Elekta Leksell Gamma Plan Version 10.1.

The mean tumor volume in our treated 22 patients was 7.26 cm^3^ (range 2.8–19.4 cm^3^). The mean tumor peripheral dose was 14.7 Gy (ranged 12–16 Gy), the mean isodose curve was 37.7 % (range 35 to 50 %), and the mean maximum tumor dose was 41 Gy (34–45 Gy), with a mean tumor radiation coverage of 96.3 % by the prescribed dose. Adjacent area of brain stem dose ranged between 10–12 Gy.

## Results

The mean age of patients was 46.8 years (range 22–72 years). There were19 females and 3 males. The tumors were located at the left side in 15 patients and right side in 7 patients (Table 1).

**Table 1:** Characteristics of the studied 22 patients undergoing GKS for glmous jugulare tumors

Parameter value	
Total no. of patients:	22
Female	19
Male	3
Age (range)	22–72 years (mean 46.8 years)
Prior intervention to GKS:	
1—microsurgical interventions	2
2—radiation therapy	2
3—embolization	3
Tumors locations:	
Left side	15
Right side	7
Glmous jugulare tumor volume:	Ranged 2.8–19.4 cm^3^ (mean volume is 7.26 cm^3^)
Follow-up period:	Ranged 36–108 months (mean is 56 ms)

The most common neurological deficit at initial evaluation was bulbar symptoms (IX, X, XI cranial nerve paresis) in 15 patients, deterioration in hearing up to hearing loss in 8, facial nerve palsy in 1 patient (previously had microsurgical resection), pulsatile tinnitus in 12 patients, and tongue deviation with wasting (XII paresis) in 1 patient. Shoulder pain, neck pain, and trigeminal symptoms also detected.

Clinical improvement was detected in 12 patients during the follow-up period, improving starting within 12 months to 24 months post GKS, and was mainly in bulbar symptoms (dysphonia, regurgitation),followed by tinnitus and also shoulder pain; 7 patients showed stable clinical condition with no additional symptoms or signs. Three patients developed new neurological deficit post gamma knife treatment, one developed transient facial nerve palsy, one developed trigeminal pain that responded to medical treatment, and one developed hearing loss in addition to facial numbness.

**Fig. 1:**
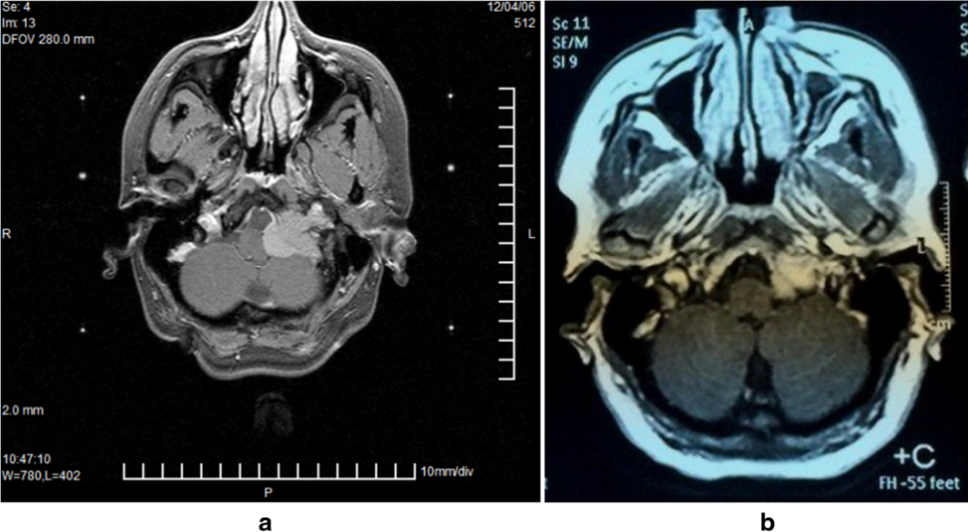
**a** Pre-gamma knife stereotactic contrast enhanced MR image of a female patient with a large 7.4 cm^3^ left glomus jugulare treated with 15 Gy marginal dose to 35 % isodose line with 98 % coverage with the prescribed dose. **b** Contrast-enhanced MR image of the same patient 96 months after GKS showing significant tumor regression

**Fig. 2:**
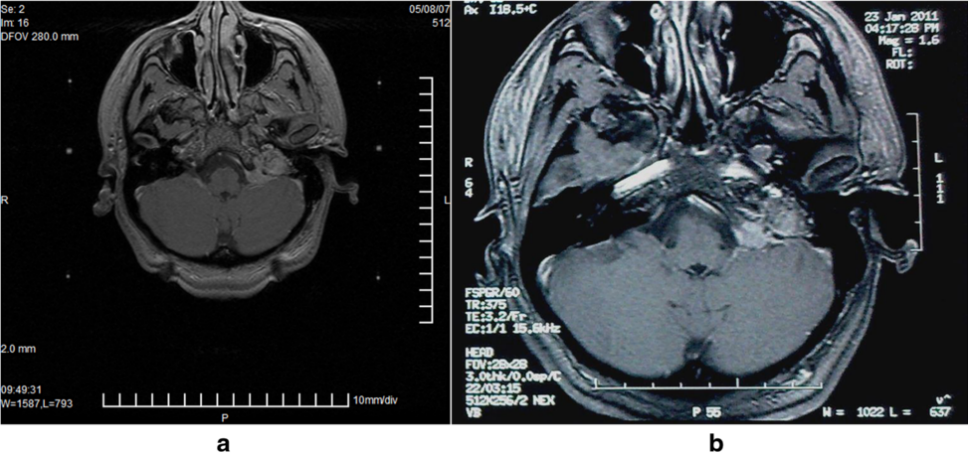
**a** Pre-gamma knife stereotactic contrast-enhanced MR image of a patient with a large 6 cm^3^ left glomus jugulare treated with 15 Gy marginal dose to 35 % isodose line with 97 % coverage with the prescribed dose. **b** Contrast-enhanced MR image of the same patient 72 months after GKS showing some tumor regression

Regarding tumor size in the follow-up MRI brain in our study 22 patients, 8 showed some tumor size reduction; the radiation coverage of their tumors was above 95 %, marginal prescribed dose was 15–16 Gy, and 13 patients had local tumor control (Figs. 1 and 2.)

One patient showed progressive tumor growth in this study (the tumor volume was 19.4 cm^3^; it was large postoperative residual and 90 % of the tumor covered by 12 Gy prescription marginal dose); the patient was retreated with gamma knife after 4 years of initial GKS treatment.

Tumor progression-free survival after GKS in our study was 95.5 % at 5 and 7 years of follow-up period.

## Discussion

Glomus jugulare tumors are slowly growing, locally destructive, highly vascular lesions located in one of the most poorly accessible surgical regions of the skull base mainly. The operative approach is, in addition, complicated by the fact that lesions may be both intradural intracranial and extradural with engulfment of critical neurovascular structures, may invade the jugular foramen, and may extend extra cranially within the upper cervical region; thus, it is not surprising that resection entails a great deal of morbidity and leaves behind usually large residual tumors [2, 3, 6, 9, 13].

Among the 22 glomus jugulare tumor patients in our series, 15 patients underwent gamma knife surgery as primary treatment, and 7 patients had GKS as adjuvant therapy. The mean age of patients was 46.8 years (range, 22–72 years); there were 19 females and 3 males.

In our study, the most common neurological deficit at initial evaluation was bulbar symptoms (IX, X, XI cranial nerve paresis) that detected in 15 patients, pulsatile tinnitus in 12 patients, deterioration in hearing in 8 patients, VII nerve palsy in 1, and tongue deviation (XII paresis) in 1 patient.

The most frequent symptoms of glomus jugulare tumors include pulsatile, tinnitus, conductive deafness, headache, migraine, hypoacusis, vertigo, and multiple cranial nerve palsy, with possible involvement of 5th to 12th cranial nerves. The neurological symptoms are related to the region affected by the tumor. This can infiltrate neurovascular structures, the temporal bone, the jugular foramen, the hypoglossal canal, the clivus, the cavernous sinus, and cervical region [1, 2, 6, 9, 14].

Green et al. showed their surgical experience with 52 previously untreated patients with glomus jugulare tumors. Lower cranial nerve preservation was possible in the majority of patients with normal preoperative function. Most patients (85 %) were able to fully resume all preoperative activities. Their results suggested that surgical management is an important option in younger patients with glomus jugulare tumors. Nevertheless, many cases postoperative have tumor residuals [7].

Gerosa et al. 2006, between 1996 and 2005, used GKS to treat 20 patients bearing growing glomus jugulare tumors with average follow-up 50, 85 months; 3 patients received GKS as primary treatment, 8 had surgical recurrences, and 11 out of the 20 patients previously underwent endovascular embolization. Neurological signs and symptoms were unchanged in 13 patients. An improvement of cranial nerve function was observed in 5 patients. For gamma knife complications, hearing deterioration was observed in 2 patients. Tumor volume was unchanged in 11 patients and was slightly decreased in 8 patients [5].

Sheehan et al. (2012) in a review under the guidance of the North American Gamma Knife Consortium, 8 gamma knife surgery centers that treat glomus tumors combined their outcome data retrospectively. One hundred thirty-four patient procedures were included in the study. Prior resection was performed in 51 patients, and prior fractionated radiotherapy was performed in 6 patients. The patients’ median age was 59 years. Forty had pulsatile tinnitus at the time of GKS. The median dose to the tumor margin was 15 Gy. The median of follow-up period was 50.5 months. Overall tumor control was achieved in 93 % of patients; actuarial tumor control rate was 88 % at 5 years post radiosurgery. Pulsatile tinnitus improved in 49 % of patients. New cranial nerve deficits were noted in 15 %; improvement in preexisting cranial nerve deficits was observed in 11 % of patients [14].

Liscak et al. (2014), during the period from 1992 to 2003, treated 46 patients with glomus jugulare tumors with the median age of 56 years. GKS was the primary treatment in 17 patients (37 %). Open surgery preceded gamma knife stereotactic radiosurgery in 46 % of cases, embolization in 17 %, and fractionated radiotherapy in 4 %. The volume of the tumor ranged from 0.2 to 24.3 cm^3^, dose to the tumor margin ranged between 10 and 30 Gy, and median follow-up period was 118 months. Neurological deficits improved in 19 of 45 patients and deteriorated in 2 patients. Tumor size decreased in 34 of 44 patients with imaging follow-up, while an increase in volume was observed in 1 patient [9].

In our studied 22 glomus jugulare patients subjected to gamma knife surgery, the median clinical and radiological follow-up period was 56 months ranged between 36 and 108 months. The mean tumor volume was 7.26 cm^3^, the mean tumor peripheral dose was 14.7 Gy, the mean isodose curve was 37.7 %, and the mean maximum dose to the tumor was 41 Gy. Clinical improvement was detected in 12 patients during the follow-up period; improvement was mainly in bulbar symptoms (dysphonia, regurgitation) followed by tinnitus and also shoulder pain; 7 patients showed stable clinical condition. Regarding post gamma knife complications, 3 patients experienced new deficits, 1 developed transient facial nerve palsy, another developed trigeminal pain that responded to medical treatment, and 1 patient developed hearing loss in addition to facial numbness

Regarding tumor size in follow-up MRI in our studied 22 patients, 8 showed some tumor size reduction, 13 had local tumor control (stable), and 1 patient showed progressive tumor growth (who had large postoperative residual of 19.4 cm^3^ volume and 90 % coverage of given 12 Gy prescription marginal dose). This patient was retreated with gamma knife 4 years after initial treatment. Tumor progression-free survival after GKS in our study was 100 % at 3 years and 95.5 % at 5 and 7 years of follow-up period.

## Conclusions

Gamma knife surgery could be used safely and effectively with limited side effects as a primary management tool in patients with glomus jugulare tumors especially those who have no significant cranial or cervical extension, in the elderly, and in patients with serious preexisting medical conditions, while it is also safe and effective as adjuvant therapy after surgical debulking and or post endovascular embolization. Despite of the limited number of the studied patients with glomus jugulare tumors (22 patients),GKS would seem to afford effective local tumor control and preserves neurological function with mean follow-up of 56 months (range 36–108 months), GKS role as primary management likely established as a valid alternative to surgery in selected patients.

## Abbreviations

GKS: gamma knife surgery
cm^3^: cubic centimeter
